# Performance-Based Financing Empowers Health Workers Delivering Prevention of Vertical Transmission of HIV Services and Decreases Desire to Leave in Mozambique

**DOI:** 10.15171/ijhpm.2017.137

**Published:** 2018-01-01

**Authors:** Roseanne C. Schuster, Octávio de Sousa, Anne-Kathe Reme, Carolyn Vopelak, David L. Pelletier, Lynn M. Johnson, Mduduzi Mbuya, Delphine Pinault, Sera L. Young

**Affiliations:** ^1^Program in International Nutrition, Division of Nutritional Sciences, Cornell University, Ithaca, NY, USA.; ^2^Center for Global Health, School of Human Evolution and Social Change, Arizona State University, Tempe, AZ, USA.; ^3^CARE Mozambique, Maputo, Mozambique.; ^4^Mailman School of Public Health, Columbia University, New York, NY, USA.; ^5^International Medical Corps, Washington, DC, USA.; ^6^Cornell Statistical Consulting Unit, Cornell University, Ithaca, NY, USA.; ^7^Global Alliance for Improved Nutrition (GAIN), Washington, DC, USA.; ^8^Department of Population Medicine and Diagnostic Sciences, Cornell University, Ithaca, NY, USA.; ^9^Department of Anthropology, Northwestern University, Evanston, IL, USA.

**Keywords:** Performance-Based Incentive, PMTCT, Motivation, Health Worker Attrition, Mozambique

## Abstract

**Background:** Despite increased access to treatment and reduced incidence, vertical transmission of HIV continues to pose a risk to maternal and child health in sub-Saharan Africa. Performance-based financing (PBF) directed at healthcare providers has shown potential to improve quantity and quality of maternal and child health services. However, the ways in which these PBF initiatives lead to improved service delivery are still under investigation.

**Methods:** Therefore, we implemented a longitudinal-controlled proof-of-concept PBF intervention at health facilities and with community-based associations focused on preventing vertical transmission of HIV (PVT) in rural Mozambique. We hypothesized that PBF would increase worker motivation and other aspects of the workplace environment in order to achieve service delivery goals. In this paper, we present two objectives from the PBF intervention with public health facilities (n=6): first, we describe the implementation of the PBF intervention and second, we assess the impact of the PBF on health worker motivation, key factors in the workplace environment, health worker satisfaction, and thoughts of leaving. Implementation (objective 1) was evaluated through quantitative service delivery data and multiple forms of qualitative data (eg, quarterly meetings, participant observation (n=120), exit interviews (n=11)). The impact of PBF on intermediary constructs (objective 2) was evaluated using these qualitative data and quantitative surveys of health workers (n=83) at intervention baseline, midline, and endline.

**Results:** We found that implementation was challenged by administrative barriers, delayed disbursement of incentives, and poor timing of evaluation relative to incentive disbursement (objective 1). Although we did not find an impact on the motivation constructs measured, PBF increased collegial support and worker empowerment, and, in a time of transitioning implementing partners, decreased against desire to leave (objective 2).

**Conclusion:** Areas for future research include incentivizing meaningful quality- and process-based performance indicators and evaluating how PBF affects the pathway to service delivery, including interactions between motivation and workplace environment factors.

## Introduction

Performance-based financing (PBF) has been cautiously embraced as a strategy to improve delivery of health services in low- and middle-income countries.^1-4^ Here we use Renmans and colleagues’ definition of PBF as “an incentive scheme directed to health providers (facilities and/or health workers), but accompanied by a new level of autonomy of the health facility (eg, to decide on the use of resources), increased monitoring, and a separation of functions between the purchaser, provider and/or the newly created verification officer of health services.”^5^ PBF purportedly works by aligning the motivation of health workers with that of the health system, a reflection of principle-agent theory.^6^ In the early decades of PBF, this definition of motivation was not challenged^7^ nor explicitly measured.^8^ However, interest in better understanding the pathway between PBF and service delivery performance was ignited by a Cochrane review reporting that variation in PBF design and lack of process analysis of these context-dependent studies made it impossible to assess general PBF effectiveness.^9^ Since then, increased ethnographic evaluation of PBF^10^ and contextual analysis has advanced our understanding of the value of each component of the PBF intervention.^5^

Improving health worker motivation is the foundation of PBF. Motivation is “an individual’s degree of willingness to exert and maintain an effort towards organizational goals.”^11^ Motivation has long been classified as intrinsic, or generated by internal forces (eg, altruism, self-improvement), versus extrinsic, or generated by forces external to the self (eg, recognition, rewards).^12^ In low-resource health systems where health workers experience low remuneration and challenging work environments (eg, poor infrastructure, weak management, high workload), extrinsic motivation is at risk of being low^13,14^ and health systems may depend upon workers’ intrinsic motivation to care for their community and a sense of duty to fulfil their role.^15^ While the ethics and sustainability of relying on intrinsic motivation, particularly in the context of task-shifting to community-based health workers, has been called into question,^16^ concern for “crowding out” health workers’ intrinsic motivation with financial incentives has been a greater concern.^17^ However, Lohmann and colleagues have proposed a conceptual framework that moves beyond the dichotomous intrinsic vs. extrinsic perspective of motivation and instead recognizes the multiple sources, origins, and regulations of motivation that influence an individual health worker.^7^ This framework is grounded in self-determination theory and fits more clearly within multidimensional PBF initiatives that simultaneously target multiple aspects of work motivation.^7^

Examination of health worker motivation in low-resource health systems prompts discussions about attrition. There is a severe shortage of skilled health workers in sub-Saharan African health systems: 20% and 10% of African-born physicians and nurses, respectively, work overseas^18^ and more have migrated elsewhere within the continent or have left the public health system for jobs with higher pay and more professional development opportunities in the non-governmental organization (NGO) or private sectors.^19^ This turnover represents not only the substantial lost return on investment of the time and money required to train and retrain workers,^20^ but also interruptions of service delivery.^21^ Job satisfaction is the positive affective orientation an employee holds towards the organization that employs them^22^ and is inversely correlated with intention to leave.^23^ Job satisfaction is strongly related to motivation^24^ and can be positively impacted by PBF initiatives.^25^ However, the impact of PBF on workers’ thoughts of leaving, desire to leave, and attrition has been understudied.

Despite recent successes in increased access to maternal antiretroviral therapy, early testing and treatment for infants and young children, and reduced incidence rates, vertical transmission of HIV still poses risk to maternal and child health, particularly in sub-Saharan Africa.^26^ In 2015, 150 000 children were infected by HIV, the vast majority through vertical transmission.^27^ Early evidence of PBF impact from the large, well-designed intervention in Rwanda has shown PBF to increase on HIV testing^28^ and key maternal and child health indicators, including quantity and quality of antenatal care and number of facility births and child preventative visits.^29^ Following this, PBF was proposed as a means to support the scaling up of preventing vertical transmission of HIV (PVT) services while motivating health workers through monetary and non-monetary incentives.^30^ A recent systematic review of impacts on HIV/AIDS services found PBF was associated with reduced patient drop-out and treatment failure but failure to clearly replicate results necessitates further validation.^31^ Challenges to implementation of national PVT policies and best practices are numerous at the point of care^32^ and are linked with delivery of the specific services critical to PVT.^33,34^ However, evaluation of the implementation of PBF interventions and the impact of PBF on intermediary constructs in the context of PVT are lacking.

We therefore tested a proof-of-concept PBF intervention with the overarching goal of increasing the number of PVT services delivered at health facilities in rural Mozambique. This paper focuses on two complementary study objectives. The first is to describe the implementation of the intervention, inclusive of incentive use. The second objective is to assess how PBF affects health worker motivation, key factors in the workplace environment, and health worker satisfaction and thoughts of leaving their position.

### Conceptual Framework

Our study design is grounded in the Motivation-Opportunity-Ability framework, which comes from the human resources and operations management literature^35^ and posits that all three of domains must be present to elicit the desired behavior to occur.^36^ We have defined motivation above and opportunity as the many contextual factors that enable or constrain the desired behavior action. Ability encompasses the skills and knowledge to execute action. Our previous work has identified that the range of factors in the opportunity domain is most limiting, and that knowledge, particularly in PVT, was not limiting^32,37^ Therefore, we focus on the intermediary workplace environment constructs as representative of the opportunity domains in addition to the motivation this is still central to PBF.

**Figure 1:**
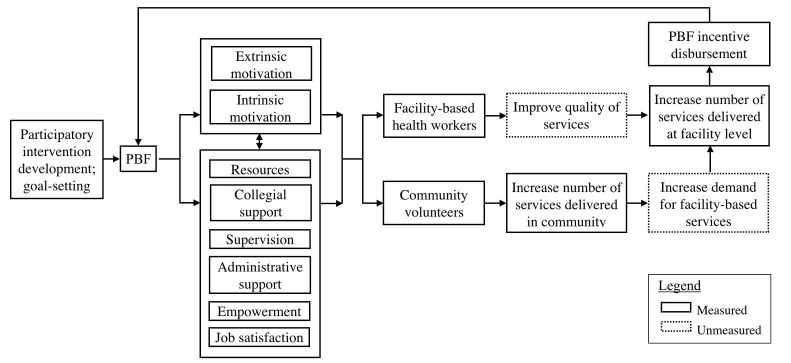
Conceptual Framework for a PBF Intervention With Facility-Based Health Workers to Increase the Quantity of Services Delivered to Prevent Vertical Transmission of HIV in Rural Mozambique. Abbreviation: PBF, Performance-based financing.

In light of this theory, we created a conceptual framework to guide the implementation and evaluation of the PBF intervention (Figure 1). Engaging stakeholders in the intervention design, specifically in goal setting, is key to effectively priming health workers’ autonomous loci of motivation^38^ and is the starting point for this intervention’s conceptual framework (Figure 1). PBF was initially hypothesized to primarily work through increasing health workers’ extrinsic motivation (eg, remuneration, social recognition) to lead to a desired action,^2^ with collective extrinsic motivation increasing collegial support towards goal achievement. However, more recent literature recognizes that the PBF package affects many other aspects of a health worker’s workplace environment, and that these are interrelated. For example, increased monitoring may lead to increased or improved supervision and administrative support^39^; increased autonomy may lead to a sense of ownership and empowerment^7^; and these and new facility resources may interact with extrinsic motivation to affect intrinsic motivation and job satisfaction.^7,25^

Our PBF intervention involved both facility-based health workers and community volunteers. We hypothesized that PBF would work through these motivation, workplace environment, and job satisfaction constructs to lead facility-based workers to improve quality of service delivery and lead to increased number of services delivered at the facility (Figure 1). Similarly, we posited that PBF would increase number of community services delivered by volunteers, including referral for facility-based care, and therefore contribute to increased demand for facility-based services. Since health workers’ experiences of prior outcomes affect effort they invest in similar situations,^11^ we expected that the effects of the PBF on workplace environment and incentives earned each quarter (including the process of disbursement) will affect how strong the PBF is as a “treatment” in the subsequent quarter.

## Methods

### Study Setting

This research was conducted from July 2012-August 2014 in two districts in northern Inhambane province, Mozambique. At that time in Mozambique, the HIV prevalence among women of reproductive age was the fifth-highest globally at 16%, women dropped out at each step of the cascade of PVT services, and 12% of children born to women living with HIV/AIDS contracted the infection through vertical transmission.^40,41^ Furthermore, Mozambique continues to experience a severe shortage of health workers,^42^ which is problematic for PVT because maternal and child health nurses and midwives provide the majority of these services.

During the period of formative work and intervention design (July 2012-July 2013),^37^ CARE International provided funding and technical support for HIV prevention and treatment for the districts as the PEPFAR-implementing partner. During intervention implementation (August 2013-July 2014), the Center for Collaboration for Health, a Mozambican NGO new to these districts, transitioned into the role of the PEPFAR-implementing partner.

### Intervention Design

The intervention district was selected based on perceived readiness by CARE and interest of the District Health Authority. Three health facilities (one large health center in the district capital, one large health center peripheral to the district capital, one small health center) were selected for implementation of the PBF intervention (Table S1, see Supplementary file 1). The comparison district was matched based on geographic and administrative similarities, and three facilities in this district were roughly matched for similarities in their catchment areas and workforce. Community volunteer associations in the two districts were also engaged in the PBF intervention,^43^ but the results are beyond the scope of this paper.

Our participatory approach to intervention design began with an 8-month formative assessment of the barriers that health workers experience in delivering PVT services and the appropriateness of PBF to address them.^37^ Following the formative assessment, repeated meetings with intervention health centers, district authorities, and health system implementing partners were held to discuss, debate, and design the intervention. The intervention district’s senior health administrator also visited another district in Mozambique with an on-going performance-based financing intervention to learn about challenges and facilitators to implementation.

### Goal Setting

The five indicators that were chosen represented all women and women living with HIV only and spanned the PVT service cascade: number of all women (1) and women living with HIV (2) attending first antenatal care visit; number of all women (3) and women living with HIV (4) delivering at health facility; and number of children exposed to HIV attending child preventative health visits (5). These indicators were suggested by the research team in design meetings with intervention district health authority and facility leaders because they are indicators used for facility- and district-level goals and reporting. The indicators were were agreed to by facility leaders, health workers, and district administrative as they aligned with current focus.

Facility-specific goals for each indicator were set by taking the monthly mean for each indicator for the previous year and then increasing it by 10%. Setting goals as a proportion of baseline measures builds on existing capacity and has been used in other results-based financing initiatives.^2^ This approach created challenging but realistic goals that allowed scaling for facilities with differently-sized patient populations.

### Incentives

The total budget for facility-based incentives for the three intervention facilities was US$18 000 or $4500/quarter. The quarterly budget was approximately ¼ of each worker’s monthly salary, so that over the year of implementation the maximum total financial incentive would be equivalent to one month’s salary for all health workers. This quarterly budget was allocated to the three intervention facilities in proportion to the number of workers and their respective salaries at the time of the intervention planning phase (Table 1). For reference, a nurse’s monthly salary at the time was US$350.

**Table 1:** Quarterly Maximum Incentive Amount Per Health Facility and the District Health Authority in the Intervention District of the PBF Intervention

**Unit**	**Number of Health Workers**	**Maximum Incentive Funds Per Quarter (US$)**
Large health center in district capital	52	$2910.00
Large health center peripheral to district capital	22	$1250.00
Small health center peripheral to district capital	4	$340.00
District health authority	5	$225.00
Total	83	$4725

Abbreviation: PBF, Performance-based financing.

An additional 5% of total health facility incentives (US$900) was allocated as incentives to engage the District Health Authority in supporting health facilities. These incentives would be awarded based upon the mean percent of goals achieved each quarter across the facilities.

### Evaluation

The five indicators were assessed quarterly in both the intervention and comparison districts, with the proportion of goals achieved based on the monthly mean for that quarter. Service delivery data were initially planned to be collected at the worker and patient levels. However, resource limitations and concerns about data completion precluded this approach and service delivery was reported at the facility level, with n = 6 health facilities.

The research team calculated proportion of goal achieved for each quarter and then held a meeting with each facility to review quarterly performance and challenges encountered. At intervention facilities, the amount of incentives earned and priorities for spending the earned incentives were also discussed.

Zero to 100% of the PBF funds were awarded based on quarterly goal achievement. Funds were to be disbursed to the District Health Authority using processes modified from CARE’s existing PEPFAR mechanisms. In order to receive the incentives, each facility was to prepare a solicitation of funds to the district and then the district administrator would generate a bank check. A justification of expenditures was required before the next quarterly transfer could occur.

### Use of Funds

Each facility created a committee with the autonomy to decide how to use the incentives earned by the facility each quarter. During intervention development, committees expressed interest in splitting earned incentives evenly between individuals and the facility. Of note, committees decided that all facility workers irrespective of job description were eligible to receive personal incentives because all staff, from the lead physician to janitor, contributed to creating a positive environment that would help retain patients in the cascade of PVT services.

### Data Collection

#### Implementation: Quantitative Service Delivery Data

**Figure 2:**
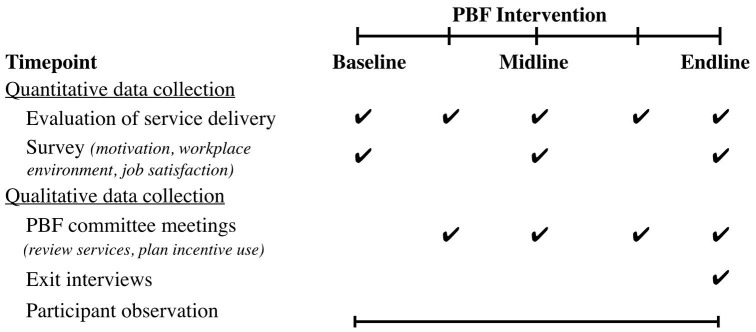
Data Collection Timeline for the PBF Intervention Implementation and Evaluation. Abbreviation: PBF, performance-based financing.

The research team sought to streamline data collection by using the existing data collection processes; the Center for Health Collaboration (the PEPFAR-implementing partner) independently checked original paper records against monthly reports submitted by health facility (Figure 2). Service delivery data were extracted from these reports, entered into Excel 2011 (Microsoft, Seattle, WA), and imported into Stata v14 (StataCorp LP, College Station, TX) for analyses.

#### Intermediary Constructs: Quantitative Motivation and Workplace Environment Surveys

*Instruments.* Motivation, workplace environment factors, and thoughts of leaving were evaluated longitudinally using a survey at intervention baseline, midline, and endline (Figure 1). Constructs from our formative work were compared to two known tools to capture health worker motivation: a questionnaire for community health workers used in Haiti and Zimbabwe^44-46^ and a tool developed to measure motivation among hospital workers in Kenya.^47^ The community health worker questionnaire used by Mbuya and colleagues captured the vast majority of constructs from our formative work and was thus selected as the basis for our survey.^44^ We modified survey language to more clearly reflect the facility-based health cadres being interviewed and the deletion of three questions that referred to home-based care (Supplementary Material 1).

The survey questions captured the constructs in our conceptual framework (Figure 1; 87 questions) as well additional constructs to monitor unintended consequences on time spent (11 questions), workload (5 questions), and other key aspects of workplace environment (eg, training, 3 questions; PVT knowledge, 15 questions). Knowledge questions were asked at baseline and endline only, and those on goals and incentives (11 questions) were asked at endline only. Questions on incentives were only asked of workers at intervention facilities. Response options were given on a 1-5 Likert-type scale (eg, never, rarely, sometimes, usually, always).

The survey was adapted to be specific to each facility worker type (eg, maternal and child health nurses, other clinicians, non-clinicians), translated into Portuguese, and back-translated into English. Experts in the local language Xitswa helped standardize the best oral translations from written Portuguese. Research assistants who conducted the surveys were native or fluent speakers in Portuguese and Xitswa and had prior experience in research or as community HIV counselors. Surveys were piloted with health workers in a similar, neighboring district not involved in the intervention and then finalized.

*Participants.* We invited all workers at the small peripheral facilities and 63% of workers at the other facilities (all maternal and child health nurses and midwives, representatives of other departments who were chosen based on departmental seniority). Only five health workers that were successfully contacted declined to participate, citing time constraints. The majority of participants filled out the surveys independently and subsequently reviewed their completed survey with a member of the research team for quality control. Others were asked the survey questions by the enumerator,

#### Implementation and Intermediary Constructs: Qualitative Data Collection

*Participant observation.* Ethnographic observation occurred at longitudinal planning meetings, quarterly performance review meetings, regular phone contact, and unstructured observation at health facilities (Figure 2). These were recorded in detailed handwritten notes in Portuguese and English. Notes were typed and compiled into an electronic notebook daily.

*Exit interviews.* Key stakeholders from both districts (eg, district health administrators, facility leaders, health workers) were invited to participate in semi-structured interviews at the end of the intervention. Participants were asked about their perceptions of the intervention in discrete steps, from design through incentive disbursement. Questions probed facilitators, challenges, and suggested improvements for the PBF intervention design and implementation (Supplementary Material 2). Interviews were conducted in Portuguese and took 55-75 minutes to complete.

### Data Analysis

#### Implementation: Quantitative Analysis of Goal Attainment

The non-parametric Wilcoxon-Mann-Whitney was used to assess differences in goal attainment status (yes, partial, no) for total proportion of goals achieved and survey questions only asked at endline. The Wilcoxon-Mann-Whitney was also used to compare demographic characteristics among survey and exit interview participants.

#### Reducing Variables Into Motivation and Workplace Environment Factors

*Intermediary constructs: Quantitative analysis.* We used factor analysis with an oblique rotation for correlated factors to reduce survey questions into salient constructs, preserving factor loadings >0.4. We made exceptions for factor loadings >0.3 if the item was strongly conceptually related to the construct. We checked the internal consistency of factors using Chronbach α >0.7 for all workers together and by intervention and comparison status, and made four exceptions for factors with Chronbach α >0.55 for all workers together in cases where the factors were strongly aligned with findings from our formative work (Table S2, see Supplementary file 1). Chronbach α can underestimate reliability and is thus considered the “lower bound” of reliability if multiple traits underlie scale items^48^ and cannot be considered an absolute confirmation of scale reliability. Composite scores for multi-item constructs were created by summing item values and dividing by the number of items.

Separate mixed effects models were used to test the impact of PBF on each of motivation, workplace environment, job satisfaction, and desire to leave constructs (objective 2) at the level of the health worker. Treatment (PBF), time, and the treatment*time interaction were modeled as fixed effects, the health facility as a random effect, and the construct as the outcome. Linear contrasts were used to test differences in construct Likert-type scores between intervention and comparison facilities between baseline and endline (objective 2).

*Post-hoc power analysis.* The sample size for the quantitative survey analysis was limited by the number of health workers at each clinic. For analysis of the psychometric variables measured on the Likert scale of 1-5, we assumed an intra-class correlation of 0.05 since workers at the same facilities may be similarly influenced by facility-level factors. Using the baseline number of health workers (intervention n = 37 and comparison n = 27), we calculated a design effect of 1.57 for intervention workers and 1.40 for comparison workers. This lead to a 60% power to detect a large effect size of 0.7.

#### Implementation and Intermediary Constructs: Qualitative Data Analysis

Audio recordings of meetings were used to augment handwritten notes. Audio recordings of the exit interviews were transcribed in Portuguese and translated into English. Two members of the research team independently coded the exit interviews using thematic analysis according to the principles of frequency, universality, differentiation, and emphasis.^49^

## Results

### Participants

Data on key PBF constructs were collected from facility health workers and district administrators through participant observation, longitudinal surveys, and exit interviews (Table 2). A total of 49 unique health workers in the intervention district and 34 unique workers in the comparison district participated in the longitudinal survey, with 55% participating in all three timepoints and 28% in two timepoints. The health workers participating in the survey were predominantly female (63%) and 30 years of age on average (Table 3). The two significant demographic differences were that in the intervention district, clinicians had a median of 9.5 more months of work experience (27.5 months vs 18 months for workers at comparison facilities) and that workers spoke one language less on average than those in the comparison district.

**Table 2:** Participants in the PBF Intervention, by Health Worker Cadre, Type of Data Collected^a^, and District

**Health Worker Cadre**	**Intervention District**			**Comparison District**		
	**Participant Observation**	**Surveys**	**Exit Interviews**	**Participant Observation**	**Surveys**	**Exit Interviews**
Health systems administrators	2	-	2	2	-	-
MCH health facility staff	18	18	2	10	10	3
Other clinical facility staff (eg, physician, *técnico,* pharmacist)	50	23	4	18	17	-
Non-clinical facility staff (eg, receptionist, cleaner, data analyst)	12	8	-	8	7	-
Total	82	49	8	38	34	3

Abbreviations: PBF, performance-based financing; MCH, maternal and child health. ^a^ Participants in participant observation represent maximum number of health workers. Of these, some participated in the surveys and some who were key stakeholders participated in the exit interviews.

**Table 3:** Demographics for All Health Facility Workers Who Participated in Longitudinal Surveys on Workplace Characteristics, by PBF Intervention Status

**Demographic Characteristic **	**Facility-Based Staff**		***P*** ** Value ** ^a^
	**Intervention**	**Comparison**	
	**(n = 47)**	** (n = 34)**	
Female	64%	62%	
Age (mean ± SD)	30 ± 9.5	29.0 ± 7.4	
Single	87%	79%	
Live with partner	36%	37%	
Have children	68%	58%	
Number of children (mean ± SD)	1.6 ± 2.4	1.2 ± 1.4	
Total number in household (mean ± SD)	4.7 ± 4.7	4.7 ± 3.1	
Number adults in household (mean ± SD)	3.2 ± 2.4	3.2 ± 2.5	
Number children in household (mean ± SD)	1.8 ± 2.5	1.6 ± 1.5	
Local origin (same or neighboring districts)	31%	25%	
Years lived in community currently serving (mean ± SD)	5.1 ± 7.0	7.4 ± 10.8	
Number languages spoken (mean ± SD)	2.9 + 1.0	3.8 + 1.1	<.001
Christian	94%	94%	
Clinical facility staff	72%	74%	
Workers providing PVTservices	36%	29%	
Education			
Primary school (1-5 years)	3%	4%	
Secondary school (6-10 years)	47%	29%	
University prep (11-12 years)	50%	68%	
Months training (mean ± SD)	25.1 + 23.4	24.6 + 14.1	
Months training (clinician only)	29.8 + 23.3	27.8 + 11.6	
Months work experience (mean ± SD)	74.3 + 100.1	49.2 + 79.3	
Months work experience (clinician)	78.9 + 110.4	36.2 + 61.9	<.01
Median (range) months of work experience (clinician)	27.5 (2, 420)	18.0 (0.25, 252)	<.01

Abbreviations: PBF, performance-based financing; PVT, prevention of vertical transmission of HIV. ^a^ *P* value refers to differences between intervention and comparison groups within the same cadre.

A total of 11 (n = 8 intervention, n = 3 comparison) health workers and administrators participated in exit interviews (Table 2). Intervention district key informants were descriptively older (mean of 31.4 vs. 23 years in the comparison) and had worked in their current position longer (mean of 6 vs. 1.5 years in the comparison). For the two facility leads in the comparison district, this was their first health position.

### Implementation of Performance-Based Financing Intervention (Objective 1)

In order to evaluate implementation of the PBF intervention, we assessed how engaged workers were in the goals setting process, the proportion of goals achieved, the process of incentive disbursement, how appropriate those incentives were, and how the incentives were used.

### Engagement in the Goal-Setting: Qualitative and Quantitative Data

Although we saw no difference in the three goal-related constructs at endline, exit interviews suggested that workers in the intervention district displayed a clearer understanding of their goals than those in the comparison. Participant satisfaction with the goal-setting and evaluation processes were mixed across both intervention and comparison facilities; some workers felt it was sufficiently participatory while others wanted more participation and a few wanted monthly (and not quarterly) evaluation.

Staff turnover created a lack of continuity in leadership that impacted intervention facilities’ abilities to implement the PBF. For example, observation revealed that the leader of the large peripheral facility who was very engaged in the PBF design and early implementation was transferred during the intervention. Similarly, after the departure of the only nurse at the small peripheral facility, a rotation of nurses and midwives staffed that facility throughout the intervention. In exit interviews, two participants described how workers who joined the facilities after the intervention had started were unclear on the goals and not as invested.

### Proportion of Goals Achieved and Amount of Incentives Earned

The intervention district fully achieved 37% of their total goal opportunities and the comparison district fully achieved 43% (Table 4). There was no difference in goal attainment for proportion of goals achieved per indicator by intervention status. Based on the proportion of fully (n = 22) and partially achieved (n = 12) goals, the intervention district earned 45.1% (US$8111) of total funds possible. The District Health Authority were awarded a corresponding 41.5% of their maximum of $900 incentives for a total of $373.5 over the 12-month implementation.

**Table 4:** Number and Proportion of Goals (n = 60)^a^AchievedAmong Health Facilities Participating in a PBF Intervention in Mozambique

**Reached Goal ** ^b^ ****	**Intervention District**				**Comparison District**			
	**Large District Health Center**	**Large Peripheral Health Center**	**Small Peripheral Health Center**	**Total**	**Large District Health Center**	**Small Peripheral Health Center**	** Small Peripheral Health Center**	**Total**
Yes	8 (40%)	7 (35%)	7 (35%)	22 (37%)	10 (50%)	8 (40%)	8 (40%)	26 (43%)
Partially	5 (25%)	5 (25%)	2 (10%)	12 (20%)	7 (35%)	3 (15%)	0 (0%)	10 (17%)
No	7 (35%)	8 (40%)	11 (55%)	26 (43%)	3 (15%)	9 (40%)	12 (60%)	24 (40%)

Abbreviation: PBF, performance-based financing. ^a^ Five goals/facility x 4 quarters = 20 opportunities to achieve goals per each health facility, and 60 opportunities to achieve goals per district across the three health facilities. ^b^ Goal is 10% increase above baseline.

### Process of Performance-Based Financing Disbursement

PBF disbursements were significantly delayed in the intervention district. There was an initial one-month delay in internal processing of incentives from CARE to the District Health Authority, followed by challenges for facility management of incentive funds, as facilities could not open a bank account. Consequently, disbursement of incentives to the large health facility in the district capital (and personal cash incentives to workers) earned for Quarters 1 and 2 occurred early in Quarter 3. The first disbursement occurred later, in Quarter 4 for the peripheral facilities, after a committee member was elected to open a personal bank account to store the PBF funds. Quarter 3 incentives arrived late into Quarter 4 for all facilities.

The most significant barrier to disbursement was leadership transitions. First, signing power was not transferred to the new financial administrator, which delayed incentive disbursements for the second and third quarters. Then, incentives to peripheral facilities were further delayed amid transitions of facility leaders and administrator vacation, which frustrated not only health workers but other administrators, as reported in the exit interviews. The small peripheral facility faced the additional challenges of remote location and lack of transportation to use incentives to purchase items for facility improvements.

### Incentive Appropriateness and Disbursement (Intervention Workers Only)

At endline, 40% of workers in the intervention district surveyed felt that the incentive amount was appropriate, and 33% were neutral. The mean response for the construct “incentives and collegial support help to improve service delivery” differed significantly by health facility (*P *= .03), with stronger agreement from workers at the small facility (4.2 ± 1.0) and slight disagreement at the other facilities (2.9 ± 1.2) on the 1-5 Likert-type scale. Incentives were delayed in reaching the health facilities, with length of delay varying by facility. At the time of the endline survey, facility-based workers at the small health center had recently received three quarters of personal incentives at one time, likely supporting their more positive response.

### Use of Incentives

**Figure 3:**
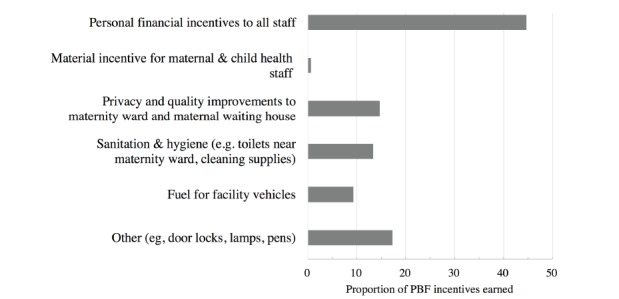
The Allocation of the PBF Funds Earned by the Three Intervention Facilities. Abbreviation: PBF, performance-based financing.

Across the three intervention facilities, half (45%) of PBF incentives earned were directed to workers as personal incentives, and the majority of the remainder was strategically allocated to incentivize women to attend the facilities for services (Figure 3). The district and large peripheral facilities opted to use incentives for infrastructure for the Quarter 1 and only allotted personal financial incentives starting in the second quarter. Across the three facilities, 15% of funds were allocated to improvements to the maternity ward (eg, privacy curtains, lamps, personal soap), maternity waiting facilities (eg, locks, electricity), and snacks for peer support groups for HIV-infected mothers. Another 13% were allocated towards improving sanitation and hygiene, including materials (eg, buckets, laundry soap) and new latrines close to the maternity ward. Fuel was used to support mobile clinics in the community, with a focus on HIV testing and antenatal care. In addition, the District Health Authority spent its incentives on iron frames for the windows to increase office security.

### Impact of PBF on Motivation, Work Environment, and Job Satisfaction and Thoughts of Leaving (Objective 2)

#### Results From Quantitative Surveys

Ten composite constructs emerged from the factor analysis of the longitudinal surveys that had acceptable internal consistency and were important to the conceptual framework (Table S2). For extrinsic motivation, satisfaction with remuneration held together as a construct but recognition from others did not, and so recognition from health workers, partners, and community were assessed as individual items. No composite construct for intrinsic motivation emerged, and so three key intrinsic motivation items (enjoys work, contributes to improving conditions in the community, improves health behaviors in a positive direction) were assessed individually. Motivating supervision and structured supervision emerged as composite constructs. Lack of resources affecting work was included for maternal and child health nurses and midwives only to reflect the focus of the intervention. Composite constructs of collegial support and training did not meet the alpha check, so two key aspects of training, adequate training and frequency of refresher training, were analyzed as individual items. Job satisfaction, feeling secure in job, frequency of thoughts of leaving, and desire to leave if other options were available were analyzed as single items. Composite and single item constructs are reported below in sections that map onto the conceptual framework (Figure 1).

#### Extrinsic Motivation

**Figure 4:**
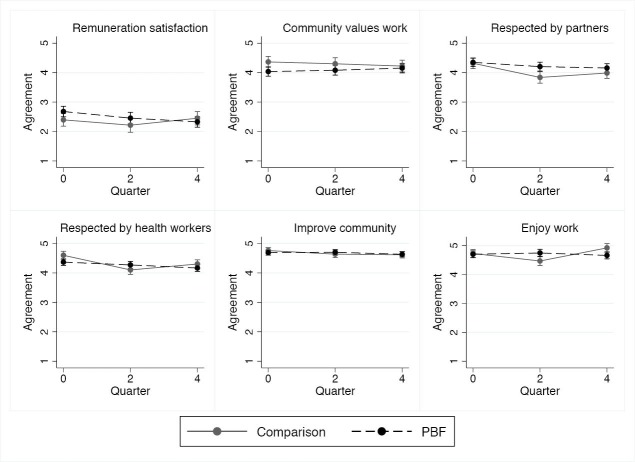
Motivation Constructs (Composite Satisfaction With Remuneration and Individual Items) as Predicted by PBF, Time, and PBF*Time Interaction for Facility-Based Health Workers in a PBF Intervention. Y-axis depicts constructs on 1-5 Likert-type scale. Abbreviation: PBF, performance-based financing.

*Satisfaction with remuneration.* At baseline, all workers were slightly dissatisfied with their compensation and there was no detectable change as a function of PBF in the longitudinal surveys (Figure 4, Table S3).

*Social recognition.* At baseline, workers strongly agreed that they were receiving respect from the community and NGO partners, and this did not change as function of the intervention (Figure 4, Table S3). However, comparison workers reported a marginally significant decrease (*P *= .08) in feeling respected by other health workers relative to intervention workers at midline.

#### Intrinsic Motivation

Health workers in both districts demonstrated high baseline levels of intrinsic motivation, as measured by three individual variables: the worker feeling he or she was contributing to improving health behaviors in a positive direction, improving conditions in the community, and enjoyment of work (Figure 4, Table S3). PBF had no detectable effect on these intrinsic motivation items.

#### Resources

**Figure 5:**
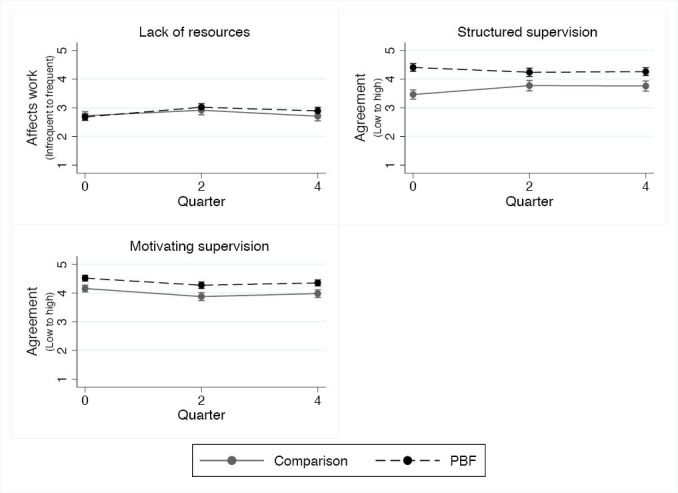
Workplace Environment Constructs as Predicted by PBF, Time, and PBF*Time Interaction for Facility-Based Health Workers in a PBF Intervention. Y-axis depicts composite constructs on 1-5 Likert-type scale. Abbreviation: PBF, performance-based financing.

Baseline quantitative surveys showed that resource shortages sometimes affected health workers’ ability to deliver care, and there was no PBF effect over the course of the intervention (Figure 5). Qualitative data from the quarterly meetings identified that during the intervention stock-outs of HIV rapid and CD4 tests, amoxicillin, paracetamol, and Nevirapine occurred.

#### Supervision

Structured and motivating supervision were higher among intervention workers at baseline. While no PBF effect was observed on motivating supervision, satisfaction with structured supervision increased among comparison workers over the course of the intervention with no change among intervention workers (Figure 5, Table S3). However, qualitative data from quarterly meetings and exit interviews shows that intervention facility and maternal and child health leaders felt that PBF led them to be more engaged supervisors and concerned about their facility’s performance.

#### Job Satisfaction and Thoughts of Leaving

**Figure 6:**
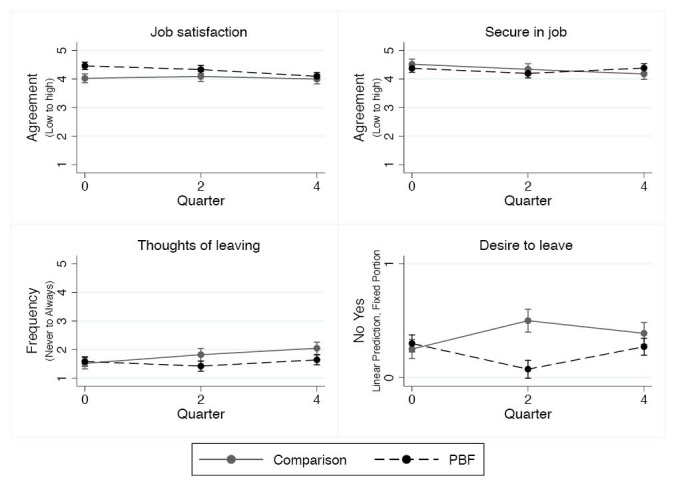
Job Satisfaction, Feeling Secure in Job, Frequency of Thoughts of Leaving, and Desire to Leave as Predicted by PBF, Time, and PBF*Time Interaction for Facility-Based Health Workers. Y-axis depicts individual items on 1-5 Likert-type scale, with the exception of thoughts of leaving (yes or no). Abbreviation: PBF, performance-based financing.

Job satisfaction and feeling secure in one’s job were high at baseline and no change was detected over the course of the PBF intervention (Figure 6, Table S3). However, there was a decrease in desire to leave if other options were available among intervention workers relative to comparison workers at midline relative to baseline (*P *= .003) and an increase in frequency of thoughts of leaving at endline but no intervention effect.

#### Additional Constructs

**Figure 7:**
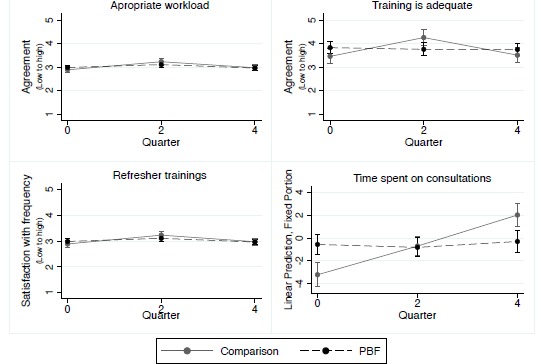
Other Workplace Environment Constructs not Central to Conceptual Framework, as Predicted by PBF, Time, and PBF*Time Interaction for Facility-Based Health Workers in a PBF Intervention. Y-axis depicts individual items on 1-5 Likert-type scale, with the exception of time spent (assigned score). Abbreviation: PBF, performance-based financing.

We found no effects of PBF on knowledge for PVT (data not shown), appropriate workload, and feeling adequately trained at endline compared with baseline (Figure 7). Workers in both districts generally felt prepared for their jobs, were neutral about their workload, and were slightly dissatisfied with frequency of refresher trainings. Time spent by nurses on antenatal and post-natal consults increased in comparison facilities but remained steady in intervention facilities.

#### Qualitative Results

Our qualitative data yielded analysis on collegial support, empowerment, and administrative support.

#### Collegial Support

In qualitative data from quarterly meetings and exit interviews, the majority of respondents from intervention facilities reported experiencing more collegial support to achieve goals compared with respondents from non-intervention facilities. An illustrative example is an intervention nurse reporting that *“colleagues were really focused on maternal and child health [sector], so they all were worried and asking*…*‘how are we doing on our goals?’”* MCH nurse, exit interview]. Workers from other parts of the facility reported increasing referrals of pregnant women to the maternal and child health sector.

In qualitative data from quarterly meetings and exit interviews, some health workers reported concern about “free riders,” or individuals who benefited from incentives but did not significantly contribute effort. In the endline survey, 19% of facility workers in the intervention district reported concern that group incentives would make some colleagues lazy and 16% were neutral. This manifested in debates for how incentives should be shared, as reported in an exit interview:

*“There were workers who received lots of complaints about their services, and when it was time to share the [incentives], some workers wanted to give less to those workers, arguing that they have a poor collaboration in the activities. They thought these workers shouldn’t receive the same amount, since some workers leave at 5 p.m. or 6 p.m. and others at 3 p.m. for instance….at the end, after an intense debate, most workers agreed that the amount should be the same for everybody…and if the workers would give stronger effort to their activities, there would be no problem”* [Facility leader, exit interview].

#### Administrative Support

The intervention facilities received mixed support from district administration. Intervention administrators were engaged in developing the PBF intervention and subsequent meetings. However, health workers reported that there was a lack of leadership in addressing obstacles that had plagued the district before and during the PBF intervention. On-going observation and reports from exit interviews identified stock-outs of medications, HIV tests, and cooking supplies for the facilities and mismanagement of fuel allotments as chronic problems.

#### Empowerment and Accountability

The PBF intervention empowered facility workers through autonomy to prioritize facility-specific issues and to address implementation barriers. During an intervention planning meeting, a facility leader reported that this was her first opportunity to be involved in prioritizing facility needs. Another facility leader noted that the group goals led health workers to “*start to see by themselves what was working or not, and what they could do to improve it in order to receive their [incentive] amount. That was a way of giving…autonomy. And that’s a good thing”* [Facility leader, exit interview].

In addition to their own engagement, PBF encouraged workers and facility leaders to look for greater accountability from district administrators. For example, health workers initiated a process that resulted in the removal of an ineffective and obstructionist administrator after that official’s actions affected the Quarter 1 of PBF earnings and disbursement. Another example was a facility leader leveraging disclosure of PBF funds used for items that should have been covered by the district, until the district administrator promised to properly allocate these items for the next quarter (Quarter 3 leadership meeting).

## Discussion

We described the process, experiences and constraints in the implementation of a PBF intervention to improve delivery of PVT services (objective 1) and evaluated the longitudinal impact of PBF on health worker motivation, workplace environment, job satisfaction, and desire to leave (objective 2). Our evaluation of this small, proof-of-concept PBF intervention revealed that implementation fidelity was compromised by administrative barriers, delayed disbursement of incentives, and poor timing of evaluation measures with incentive disbursement. At the facility level, we found that PBF increased collegial support, worker empowerment, and decreased desire to leave in a time of transitioning funding and implementing partners.

Our analyses adds to the growing literature on the importance of careful PBF implementation and its evaluation in parsing out intervention effects. We confirmed that increased autonomy in deciding how to use funds was motivating and contributed to workers’ empowerment, which has similarly manifested among hospital management in Rwanda,^50^ facility workers in Nigeria,^51^ and supply chain workers in Mozambique.^52^ However, the challenges encountered when implementing a new and different system such as PBF suggest that significant time is needed to streamline processes. For example, the delay in incentive disbursement in this intervention might have been resolved over a longer implementation period, as well as with more intensive up-front investment. Elsewhere in Mozambique, a large-scale PBF intervention took 1.5 years of implementation to begin to see impacts of PBF on PVT service delivery indicators.^53^ Smooth implementation of PBF interventions are important, as PBF evaluations in Uganda and Tanzania suggest that deviations from planned implementation that lessen the “dose” of the intervention and may negatively affect the motivational pathway.^54,55^

This study’s most valuable finding may be the ways in which the PBF led facility workers to demand accountability from the health system administration, impacting both implementation and workplace environment. Health worker participation in design and implementation of PBF initiatives such as this one contributes to feelings of ownership and fulfillment of the multiple dimensions of health worker motivation including its autonomous source, feeling valued, and satisfaction with demonstration of competence.^7^ This suggests that evaluating the multiple dimensions of motivation, specifically the ones reinforced by the autonomy and empowerment that can be introduced by PBF packages, has the potential to lead health workers to challenge some systemic barriers that otherwise are somewhat accepted as the status quo. Health workers advocating for change from the bottom-up supports the potential for broadening functional leadership in highly centralized and hierarchical health systems.^56^ This is a self-reinforcing feedback look, as autonomous sources of motivation are posited to lead to increases in worker performance in the context of PBF.^6^

Our findings of increasing collegial support towards goal achievement for intervention workers and the increase in structured supervision received by comparison workers adds to the growing body of literature on the PBF package strengthens health systems. PBF success in the Mozambican supply chain was due, in part, to enhanced teamwork.^52^ However, concerns about non-contributing health workers benefitting from the personal financial incentives rewards remains problematic, in our study and in the literature. This “free riding” is a commonly cited source of concern for PBF interventions,^53^ although contrary evidence has also been reported.^54^ Increased supervision that comes as part of the package of PBF interventions directly motivated health workers in Benin^39^ and were a source of pride for health workers in Nigeria.^51^ The additional monitoring that comes with PBF evaluation may have led to the observed increase in satisfaction with structured supervision among comparison workers, with no increase among intervention workers who were already well-satisfied. This in turn may have contributed to increased completeness of services per consult, which was unmeasured, and contributed to the increase in amount of time spent per consultation among comparison workers at endline compared to baseline.

Furthermore, our findings add to the limited literature of PBF impacts on attrition. PBF has been shown to decrease attrition among nurses and administrators in Zambia.^23^ While we recognize the conceptual distinctions between frequency of thoughts of leaving, intention to leave, and attrition, we believe our findings that PBF buffered against increased thoughts of leaving is valuable in the context of the severe health worker shortage in Mozambique.

We did not observe the positive impacts hypothesized in other parts of our conceptual framework, including extrinsic or intrinsic motivation. This may be because motivation was high among both intervention and comparison workers. However, careful attention to timing of evaluation measures with respect to incentive disbursement may better capture impact of incentives on motivation and workplace environment constructs. Due to delays in incentive disbursements and focus on maintaining the planned timeline of midline and endline assessments, we may have missed changes in extrinsic motivational or satisfaction with goal attainment. Timing quantitative and qualitative assessments to assess constructs prior to and after review of achievement and incentive disbursement may help to isolate the impacts of PBF on these constructs, and give a clearer picture of how these constructs interact to impact healthcare delivery.

This paper sought to respond to calls in the PBF literature for theory-based, mixed methods evaluations to generate more knowledge of the pathway by which PBF impacts health worker performance. Recent evaluations of innovative financing initiatives have moved the field towards this goal with inclusion of the PBF conceptual frameworks and incorporation of key qualitative insights.^54,55,57^ We have learned a number of lessons in the implementation and evaluation of this PBF intervention that we believe are pertinent to advancing the field.

Through our evaluation, it became clear that measuring quantity of services delivered would not necessarily capture changes in health worker performance in this PBF intervention, if indeed present. We had chosen to incentivize *quantity* of services delivered based on their importance in capturing women’s movement through the cascade of PVT services and their widespread and successful use in early large-scale, PBF interventions.^29,58^ However, our evaluation of this small-scale pilot underscored how the service delivery indicators were not proximally responsive to the incentives, that too many intermediary steps in our conceptual framework remained unmeasured, and no impact was observed on other hypothesized intermediaries. For example, the number of PVT services delivered relies heavily on maternal attendance at health facilities, which is influenced by a multitude of opportunity challenges in the woman’s life unrelated to care delivered by health workers. And while two of the facilities directed PBF funds to items that would incentivize women’s uptake of facility care (eg, privacy curtains, soap, electricity, etc), effects of these incentives on maternal attendance would not be immediate and were further exacerbated by administrative delay.

Instead, *meaningful quality* and *process* indicators may have been more appropriate. Quality indicators are increasingly used in PBF interventions as the incentivized indicators or as weights applied to quantity-focused goals to determine final PBF fund values.^50,58-60^ Process indicators that may help to build accountability^52^ may more directly capture health workers’ performance if they are in areas within health worker control. In the context of PVT, better indicators to incentive may have included quality or completeness of counseling for adherence to medications, care during delivery, counseling on infant and young child feeding, and linkage with community-based care. However, attention must be paid to ensure these process and quality indicators are meaningful and will contribute to improved process and clinical outcomes, as opposed to the structural quality indicators that are commonly employed in PBF evaluations (eg, latrine and fence presence and upkeep, timely reporting).^61^

## Strengths and Limitations

Our study strengths included a basis on a theoretical conceptual framework, participatory design approach, and mixed methods evaluation. Another strength was the implementation of the PBF intervention in health facilities that reflects the current health system by: using existing national health system and PEPFAR infrastructure, investing very little prior to the intervention, and working on a modest budget compared with PBF initiative budgets 3-4 magnitudes greater.^39,50,58^ Therefore, the intervention was implemented in the low-resource facilities typical of the system in which most Mozambican women receive PVT care and illustrates positive intervention effects and the implementation issues that require attention in such situations. Finally, we documented how earned incentives were allocated by facility committees in ways intended to improve quality and facilitate retention of women in the cascade of care. This is an important part of implementation as incentive use can have time lag effects.

We nonetheless had important limitations. In this pilot study, we prioritized choosing a comparison district demographically and geographically similar to that of the intervention and matched individual facilities on size and patient volume, but still intervention clinicans had more years of work experience than clinicians in the comparison district. As years of nursing experience is associated with greater empowerment among nurses,^62^ intervention health workers may have contributed to a facilitity environment primed for operational autonomy and leadership in respect to comparison facilities with workers who more recently completed training. This may have contributed to the positive effects of PBF on worker empowerment.

Our proof-of-concept study was limited by the small number of health facilities (n = 6) and the corresponding number of workers (total n = 116, surveyed n = 83). The resultant low power for the survey data (60% for a large effect size of 0.7) meant that we were unable to look at how the constructs interacted with each other on the pathway to service delivery, and that we may have been unable to detect small and medium effects. However, the values from the Likert-type reponses for the non-significant constructs (eg, intrinsic motivation, extrinsic motivation, lack of resources, motivating supervision, job satisfaction) are similar between intervention and comparison workers so that even a significant effect would not necessarily be relevant in a service delivery context.

The survey that measured health worker motivation and workplace constructs was adapted from a survey used with community health workers. While this survey reflected constructs that we had identified in our formative work, it may not have best captured the ways in which PBF impacted these. Low Chronbach α for some of the composite constructs (eg, intrinsic motivation, collegial support) led to their non-use in longitudinal modeling in favor of individual items. For future studies, we recommend recognition of the multidimensional nature of motivation into our quantitative tools and move beyond the intrinsic vs. extrinsic dichotomy. While our evaluation did not detect distortions – eg, items of intrinsic motivation and social recognition started and remained high among workers in both districts and satisfaction with compensation started low and remained somewhat low - this may reflect the low power of our analyses.

Finally, due to resource limitations the data were not independently validated by checking with service recipients. Although PBF introduce concerns about gaming and manipulation of reporting,^63^ we believe this bias was unlikely since we did not see a difference in goal attainment by PBF treatment. Furthermore, recent scholarship has shown that community verifications generate unintended consequences affecting the work environment of the verifiers, loss of patient confidentiality, and inter-personal strife.^64^

## Conclusion

Evaluation of this proof-of-concept PBF implementation and its impact on intermediary constructs to service delivery demonstrate that PBF affects key aspects of the workplace environment, including collegial support, worker empowerment, and desire to leave. A large-scale study design that evaluates the interactions of motivation and intermediary workplace constructs can help to better ascertain how PBF leads to changes in service delivery, and ultimately, health impact. Incentivizing a combination of meaningful process, quality, and service indicators that are more proximally responsive to health worker behavior may help to elucidate how PBF impacts the pathway to service delivery.

## Acknowledgements

We are grateful to the frontline health workers and district health authority, who provide critical services to women and children on a daily basis, for their support and participation. We thank Cassimo Faquiral, Francisca Pascoal, Xadreque Vilankulo, and Susana Huo for conducting the longitudinal surveys. We thank Adeniyi Giwa and Juliet Lyon Edwards for strategic support in study design; Deima da Mirela, Alexandre Mupetse, and Pedro Sigano for logistical support throughout the intervention; and Emily Martey, Devon McMahon, Paige Killelea, and Josh Miller for assistance in data cleaning and analysis. We greatly appreciate the comments of the three anonymous reviewers which strengthened our manuscript.This research was funded by the Atkinson Center for a Sustainable Future through the Impact for Innovation Fund (SLY and DP) and supplemented with funding from the Mario Einaudi Center for International Studies (RCS) and the Cornell Graduate School (RCS). SLY was supported by the National Institute of Mental Health K01 MH098902.

## Ethical issues

Ethical approval was granted by the Cornell University Institutional Review Board for Human Participants (Protocol ID# 1205003043), and letters of support were obtained from the district and provincial health authorities in Mozambique. Informed written consent was obtained from each participant for surveys at each timepoint and for all exit interviews. Group oral consent was obtained at the beginning of meetings.

## Competing interests

Authors declare that they have no competing interests.

## Authors’ contributions

RCS, DP, and SLY were responsible for study conception, design, administrative and technical support, and obtaining funding. RCS led data acquisition, statistical analysis, and interpretation and manuscript drafting and critical revisions. SLY supported analysis and interpretation and critical revision of the manuscript. OS, AKR, and CV were key in acquisition of data and administrative and technical support. LMJ provided statistical support. MM and DLP provided technical support and critical revision of the manuscript.

## Authors’ affiliations

^1^Program in International Nutrition, Division of Nutritional Sciences, Cornell University, Ithaca, NY, USA. ^2^Center for Global Health, School of Human Evolution and Social Change, Arizona State University, Tempe, AZ, USA. ^3^CARE Mozambique, Maputo, Mozambique. ^4^Mailman School of Public Health, Columbia University, New York, NY, USA. ^5^International Medical Corps, Washington, DC, USA. ^6^Cornell Statistical Consulting Unit, Cornell University, Ithaca, NY, USA. ^7^Global Alliance for Improved Nutrition (GAIN), Washington, DC, USA. ^8^Department of Population Medicine and Diagnostic Sciences, Cornell University, Ithaca, NY, USA. ^9^Department of Anthropology, Northwestern University, Evanston, IL, USA.

## Supplementary files


Supplementary file 1 contains Tables S1-S3, Supplementary Material 1, and Supplementary Material 2.

## Key messages

**Implications for policy makers**

- The collective goals and incentives associated with performance-based financing (PBF) led to improved teamwork and empowered health workers to overcome systemic barriers, although care must be taken to address non-contributors.
- PBF interventions should incentivize meaningful quality, process, and service indicators that reflect behaviors within
- Well-timed longitudinal mixed methods evaluation of impact of PBF on and interactions between workplace factors are necessary to better ascertain how PBF leads to changes in service delivery.

**Implications for the public**

Health workers in low-resource settings are critical in delivering healthcare to vulnerable populations. Mechanisms to support worker motivation, engagement, and retention are important to the functioning of these health systems, and financing based on incentivizing worker and health system performance is one such mechanism. We found that in rural Mozambique, performance-based financing (PBF) led to increased teamwork and empowerment and buffered against desire to leave. Careful implementation of well-monitored PBF interventions has the potential to encourage health workers to more deeply engage with their colleagues, patients, and workplace.
