# Supplementary material for: Performance-Based Financing Empowers Health Workers Delivering Prevention of Vertical Transmission of HIV Services and Decreases Desire to Leave in Mozambique
Source: Int J Health Policy Manag. 2018 Jan 1;7(7):630–44. doi: 10.15171/ijhpm.2017.137 (PMC6037490; doi:10.15171/ijhpm.2017.137)
Supplement: Supplementary file 1 — contains Tables S1-S3, Supplementary Material 1, and Supplementary Material 2. [file ijhpm-7-630-s001.pdf]

Table S1. Demographic and Health Service Characteristics of Catchment Areas for Intervention and Comparison Facilities Prior to Implementation of Performance-Based Financing Intervention

	**PBF (Intervention) District**			**Comparison District**		
	**Large District Health Center**	**Large Peripheral Health Center**	**Health Center**	**Large District Health Center**	**Health Center**	**Health Center**[a]
**Demographics**						
Population in catchment area[b]	22,296	25,083	2,899	7,904	7,303	
Population of women of reproductive age in catchment area[b]	4,437	4,992	577	1,573	1,461	
Number women eligible for prenatal care[b]	1,115	1,254	145	395	367	
Monthly mean all pregnant women new to prenatal care (July 2012-Jun 2013)	85.9	53.7	11.6	56.5	16.4	4.5
Estimated monthly mean number HIV-infected pregnant women eligible for PTV care[c]	117.1	131.7	15.2	37.5	34.9	

Monthly mean HIV-infected pregnant women new to prenatal care (July 2012-Jun 2013)	17.8	10.5	1.5	10.9	2.3	0.8
**Health facility services**						
Measures CD4	Yes	Yes	No	Yes	No	No
Offers antiretroviral prophylaxis	Yes	Yes	Yes	Yes	Yes	Yes
Offers antiretroviral therapy (ART)	Yes	Yes	No	Yes	No	No
Maternity services	Yes	Yes	Yes	Yes	Yes	Yes
**Health care workers in catchment area**						
Facility-based staff<br><br>MCH = maternal and child health	52 staff including:<br><br>2 doctors, 5 technical clinicians, 9 general and **4 MCH nurses**, **4 midwives**, 2 HIV counselors, 1 data analyst, 9 janitors	22 staff including: 4 technical clinicians, 2 general nurses, **6 MCH nurses**, 2 HIV counselors, 1 data analyst, 4 janitors	4 staff including: 1 general nurse, **1 MCH nurse**, 1 pharmacist, 1 janitor	30 staff including: 2 doctors, **3 MCH nurses**	4 staff including: 1 technical clinician, 1 nurse, 1 **MCH nurse**, 1 janitor	2 staff including: **1 MCH nurse**, 1 janitor
# clinical staff per 10,000 population	10.8	4.8	6.9		4.1	
Community volunteers	33	3	2	10	0	0

Community health workers	8	10	2	4	4	2
Traditional birth attendants	10	2	0	2	6	1

Abbreviation: PBF, performance-based financing.

[a] Data missing for this Type II peripheral facility. [b] Based on Ministry of Health data and goals for each health facility. [c] Number of women eligible for prevention of vertical transmission services based on estimate of 10.5% HIV prevalence among women of reproductive age in PBF intervention district and 9.5% in the comparison district.

Table S2. Workplace Constructs for Facility-Based Health Workers Confirmed by Factor Analysis, by Internal Consistency (Chronbach α) for All Workers and by PBF Intervention Status

			**Chronbach α**			
**General Construct**	**Summary Description**	**Number of Variables**	**All Workers**	**Intervention Workers**	**Comparison Workers**	**Number of Variables Excluded by α**
**Constructs in PBF pathway, measured at baseline, midline, endline**						
Satisfaction with compensation	Satisfied with compensation relative to comparable jobs, responsibilities, colleagues	3	0.84	0.94	0.71	0
Motivating supervision	Supervisor inspired by example and interactions with worker, valued worker opinions	5	0.77	0.74	0.76	2
Structured supervision	Supervisor provided structure, feedback on records, updates	4	0.66	0.65	0.70	0
Lack of resources affects work	Lack of medications (ARVs, ART, other), PCR tests, supplies for reporting, transportation	7	0.73	0.80	0.72	0
**Constructs in PBF pathway, measured at endline only**						
Structured goals and recognition	Team is capable and receives recognition, deadlines and pressure help to achieve goals	6	0.81	0.76	0.87	0
Reasonable goals focused workers	Workers participated in goal-setting, goals were	4	0.63	0.60	0.67	0

	reasonable and focused workers					
Goals created paths to achievement	Colleagues and action plan helped reach reasonable challenging goals	3	0.55	0.68	0.50	0
Incentives[a]	Individual incentives and collegial support helped improve service delivery, make health workers feel recognized	3	-	0.79	-	0
**Constructs monitoring potential negative effects at baseline, midline, endline**						
Time spent[b.c]	All antenatal and HIV-exposed child consults	4	0.79	0.79	0.79	0
Appropriate workload	Amount of work for appropriate for quality expectations and compared to colleagues; time demands and pressure are manageable	5	0.62	0.66	0.54	1

[a] These questions were asked of the intervention group only.
[b] These task-specific questions were only asked of maternal and child health nurses and midwives.
[c] Principal component analysis was used to generate a score.

Table S3. Difference Between Health Workers' Motivation, Satisfaction with Workplace Environment, and Thoughts of Leaving at PBF and Comparison Health Facilities

PBF vs. comparison		Extrinsic motivation: Remuneration	Extrinsic motivation: Community values work	Extrinsic motivation: Respected by partners	Extrinsic motivation: Respected by health workers	Intrinsic motivation: Improved health behaviors	Intrinsic motivation: Improved community conditions	Intrinsic motivation: Enjoys work	Resources (clinical staff only)	Supervision: Structured	Supervision: Motivating
Intervention											
	Effect	0.284	-0.329	0.030	-0.233	0.103	-0.068	-0.027	-0.041	0.945***	0.362*
	Std. error	0.281	0.242	0.230	0.174	0.151	0.131	0.181	0.189	0.208	0.157
Time 2											
	Effect	-0.180	-0.065	-0.482*	-0.497**	-0.150	-0.121	-0.260	0.193	0.311+	-0.282+
	Std. error	0.274	0.226	0.222	0.182	0.146	0.141	0.196	0.188	0.185	0.157
Time 4											
	Effect	0.056	-0.142	-0.332	-0.297	-0.064	-0.143	0.194	-0.017	0.296	-0.176
	Std. error	0.271	0.227	0.222	0.181	0.147	0.140	0.193	0.191	0.190	0.159
Intervention*Time 2											

	Effect[a]	-0.044	0.109	0.338	0.402+	-0.204	0.131	0.305	0.144	-0.479*	0.036
	Std. error	0.352	0.291	0.279	0.232	0.187	0.183	0.251	0.239	0.237	0.201
Intervention*Time 4											
	Effect[a]	-0.406	0.260	0.140	0.098	-0.042	0.086	-0.233	0.229	-0.444+	0.010
	Std. error	0.345	0.294	0.280	0.231	0.188	0.180	0.248	0.243	0.241	0.203
N		156	160	159	164	164	162	164	120	165	164

+*P* <.10 **p*<.05 ** *P* <.01 *** *P* <.001

[a] Effect and standard error have the same values as contrast and standard error for corresponding timepoints.

Supplementary Table 3. *Continued*

PBF vs. comparison		Job satisfaction	Secure in job	Frequency of thoughts of leaving	Desire to leave	Workload	Adequate training	Refresher training	Time spent
Intervention									
	Effect	-0.567*	-0.142	0.065	0.049	0.097	0.369	0.227	2.654
	Std. error	0.223	0.226	0.254	0.111	0.162	0.406	0.305	1.255
Time 2									
	Effect	-0.215	-0.179	0.302	0.249*	0.343*	0.797**	0.061	2.513***
	Std. error	0.227	0.224	0.235	0.123	0.171	0.300	0.295	0.707
Time 4									
	Effect	-0.125	-0.342	0.527*	0.138	0.087	0.051	0.309	5.235***
	Std. error	0.222	0.228	0.243	0.121	0.170	0.301	0.299	0.898
Intervention*Time 2									
	Effect[a]	0.346	-0.002	-0.464	-0.475**	-0.220	-0.868*	0.091	-2.767**
	Std. error	0.293	0.284	0.305	0.159	0.221	0.382	0.373	0.896

Intervention*Time 4									
	Effect[a]	0.495+	0.350	-0.470	-0.166	-0.112	-0.133	-0.483	-4.976***
	Std. error	0.288	0.286	0.310	0.157	0.219	0.382	0.375	1.111
	N	167	153	162	161	167	164	159	23

+ *P*<.10 **P* <.05 ** *P* <.01 *** *P* <.001

[a] Effect and standard error have the same values as contrast and standard error for corresponding timepoints.

Supplementary Material 1

Survey questions that have been used to in composite constructs and as single items to represent constructs when the composite was not reliable

**Construct**	**Question**
Extrinsic motivation: Satisfaction with salary	c9, c10, c11
Extrinsic motivation: Respected by partners (NGOs)	c15
Extrinsic motivation: Respected by other health workers	c16
Extrinsic motivation: Community values work	c14
Intrinsic motivation: Improving community	c1
Intrinsic motivation: Enjoys work	b1
Resources	e1hivmed, e2prop, e2bomed, e3rapid, e4pcr, e5stationary, e7atransp
Supervision: Structured	d9, d10, d12, d22
Supervision: Motivating	d15, d16, d17, d18, d19
Job satisfaction	c19 (reverse coded)
Secure in job	c13
Frequency of thoughts of leaving	b12
Intention to leave	c20
Appropriate workload	b4r, b13r, c6r, c17, c18r, f2
Adequately prepared	b3
Adequate training	c3
Referesher training	c4

Time spent (principal component analysis)	g3a_1firstanc g3b_1fuanc g3c_1fuanchiv g3e_1ccr
**Asked only at endline**	
Structured goals and recognition	i3, i5, i9, i16, i17(reverse), i19
Reasonable goals focused workers	i2, i5, i7, i21
Goals created paths to achievement	i2, i13, i15
Incentives	j7, j10, j11

Q. No	From interviews with *[type of health worker],* they told us about some of the things that motivate them to do their jobs well. The following statements are examples of the things that they told us. For each of these statements, could you please indicate **HOW OFTEN** you personally, have felt this way **–ALWAYS, OFTEN, SOMETIMES, RARELY or NEVER.**<br><br>*Only indicate one response for each declaration. If the declaration is not applicable (N/A), circle 0.*						
		**Always**	**Often**	**Sometimes**	**Rarely**	**Never**	**N/A**
B1	I feel like I enjoy my work as *[type of health worker*].	5	4	3	2	1	0
B2	I feel that my work will improve people's lives.	5	4	3	2	1	0
B3	I feel like I am adequately prepared for my responsibilities.	5	4	3	2	1	0
B4	I feel like I am responsible for so many activities that it's not possible to do them all well in the time I have.	5	4	3	2	1	0
B5	I feel connected with my colleagues.	5	4	3	2	1	0
B6	I feel supported by the other *[type of health worker*].	5	4	3	2	1	0
B7	If I were sick, I could easily find someone to help with my *[type of health worker*] duties.	5	4	3	2	1	0
B8	I trust the advice I receive from colleages.	5	4	3	2	1	0
B9	There is someone who gives me information to help me understand a *[type of health worker*] work's situation.	5	4	3	2	1	0
B10	There is someone I work with who I can ask advice about handling family problems.	5	4	3	2	1	0

B11	If I need help visiting patients I know that I can always get help from the other *[type of health worker*].	5	4	3	2	1	0
B12	I have had thoughts of leaving this job.	5	4	3	2	1	0
B13	I feel that I am responsible for more work than other colleagues.	5	4	3	2	1	0
B14	I am proud to tell others that I am a *[type of health worker*].	5	4	3	2	1	0
B15	I believe that the majority of people would promote community health and put extra effort into doing so without receiving any remuneration. *(Rephrased)*	5	4	3	2	1	0
B16	Effort at work is directly proportional to what I receive.	5	4	3	2	1	0
B17	I arrive to work on time.						
B18	I belong to a formal professional organization.	(1) Yes (2) No					
B19	The professional association that I belong to influence my professional practices.	(1) Yes (2) No (3) Somewhat (0) N/A					

C. JOB MOTIVATION AND SATISFACTION

Q. No.	The following statements are also examples of the things that health workers said motivated them. This time, for each of these statements, could you please indicate HOW STRONGLY you, personally, AGREE with the statement? Do you **STRONGLY AGREE, AGREE, NEUTRAL, DISAGREE, OR STRONGLY DISAGREE**. *Only indicate one response for each declaration. If the declaration is not applicable (N/A), circle 0.*					
		**Strongly Agree**	**Agree**	**Neutral**	**Disagree**	**Strongly Disagree**	**N /A**
C1	I am contributing to improving the conditions of the communities I am working in.	5	4	3	2	1	0
C2	I help to change behaviors in a positive direction.	5	4	3	2	1	0
C3	I received adequate training to meet my current responsibilities.	5	4	3	2	1	0
C4	I feel like I receive refresher training as often as I need it.	5	4	3	2	1	0
C5	I am involved personally in my job.	5	4	3	2	1	0
C6	I have a lot of pressure in this job. It really seems like the workload keeps increasing.	5	4	3	2	1	0
C7	I find my job to be motivating and I like to do it.	5	4	3	2	1	0
C8	I feel that the health workers in the communities (matronas, activistas, APEs) value the work that I do.	5	4	3	2	1	0
C9	I am satisfied with my allowance compared with other jobs in the same area.	5	4	3	2	1	0

C10	I am satisfied with the allowance I receive for the work I do.	5	4	3	2	1	0
C11	I am satisfied with my allowance when I compare it to that of others who have backgrounds and experience comparable to mine.	5	4	3	2	1	0
C12	Overall, I am satisfied with my job when I consider the expectations I had when I became a *[type of health worker].*	5	4	3	2	1	0
C13	I feel secure that I will not lose my job in the near future.	5	4	3	2	1	0
C14	The community values our efforts to improve their lives.	5	4	3	2	1	0
C15	Partners and Organizations, such as NGOs, respect the work that I do.	5	4	3	2	1	0
C16	The other types of health professionals respect the work that I do.	5	4	3	2	1	0
C17	I am happy with the amount of time I dedicate to my job as a *[type of health worker*].	5	4	3	2	1	0
C18	My work as a *[type of health worker]* gets in the way of my work at home.	5	4	3	2	1	0

C19	Overall, how would you say you you feel with your job? Tell me if you are very satisfied, satisfied, neutral/undecided, somewhat dissatisfied, or very dissatisfied.<br>*(CIRCLE ONE RESPONSE)*	Very satisfied .... 1<br>Satisfied .... 2<br>Neutral/Undecided......................... 3<br>Dissatisfied .... 4<br>Very dissatisfied……………… 5
C20	If it were possible or if you had other alternatives, would you want to leave this job?	No .... 0<br>Yes 1

D. SUPERVISION

D0	**Now we are going to talk about your supervisor. Do you have a supervisor? Who is it?**	______________________<br>*(Write the title of the person, not their name)*

	Some health works have a good experience with their supervisor, others have problems. The following statements are intended to reflect the various roles that a supervisor at the health facility has. For each of the statements, please think about your direct supervisor at the health facility and indicate HOW OFTEN you have experienced each of the following behaviors with your supervisor. Tell me if you experience this **ALWAYS, OFTEN, SOMETIMES, RARELY, or NEVER.**<br><br>**Again, please be reassured that all your responses are CONFIDENTIAL and none of the supervisors will have access to your responses.**<br><br>*Only indicate one response for each declaration. If the declaration is not applicable (N/A), circle 0.*

		Always	Often	Sometimes	Rarely	Never	N/A
D1	My supervisor gives me information following-up to my concerns/worries	5	4	3	2	1	0
D2	My supervisor respects my fixed monthly activities when planning other meetings.	5	4	3	2	1	0
D3	My supervisor takes my concerns into account when planning activities that involve me.	5	4	3	2	1	0
D4	When I make a mistake on the job, my supervisor scolds me.	5	4	3	2	1	0
D5	My supervisor praises me when I do something really well.	5	4	3	2	1	0
D6	I feel that my supervisor takes my concerns up to the higher level of supervision.	5	4	3	2	1	0

D7	My supervisor uses times when I make mistakes or don't perform well as opportunities to help me improve my skills.	5	4	3	2	1	0
D8	I know who to approach when I encounter challenges in my work.	5	4	3	2	1	0
D9	My supervisor inspects the registers that I use.	5	4	3	2	1	0
D10	My supervisor gives me feedback on the registers that I use.	5	4	3	2	1	0
D11	My supervisor helps me to organize my time and activities in an efficient manner.	5	4	3	2	1	0

D. SUPERVISION *(Continued)*

	The following statements are intended to reflect the various roles that a supervisor has. For each of the statements, please think about your direct supervisor and indicate HOW STRONGLY each of the statements captures the behavior or attitude of your supervisor. Please tell me if you **STRONGLY AGREE, AGREE, NEUTRAL, DISAGREE, OR STRONGLY DISAGREE.** *Only indicate one response for each declaration. If the declaration is not applicable (N/A), circle 0.* **Again, please be reassured that all your responses are CONFIDENTIAL and none of the supervisors will have access to your responses.**							
		**trongly gree**	**Agree**	**Neutral**	**Disagree**	**Strongly Disagree**	**N/A**	
D12	I feel well informed by my supervisor about changes/ modifications to the activities that I am involved in.	5	4	3	2	1	0	
D13	I see my supervisor as often as I need to.	5	4	3	2	1	0	

D14	My supervisor is easy to talk with	5	4	3	2	1	0	
D15	When I disagree with my supervisor I feel comfortable to express my opinion.	5	4	3	2	1	0	
D16	Having a supervisor visit me motivates me to do a better job.	5	4	3	2	1	0	
D17	The way the supervisor provides feedback on my performance at work inspires me to do/be my best	5	4	3	2	1	0	
D18	My supervisor’s commitment to her job motivates me to do my best.	5	4	3	2	1	0	
D19	My supervisor takes into account/considers my suggestions to improve things	5	4	3	2	1	0	
D20	I feel that my supervisor is sympathetic to my problems and cares about my problems	5	4	3	2	1	0	
D21	I constantly learn new things about maternal and child health from my supervisor (technical information)	5	4	3	2	1	0	
D22	My supervisor gives me enough guidance and structure to help me do my job.	5	4	3	2	1	0	
D 23	My supervisor ensures me that I have the resources that I need to do my job	5	4	3	2	1	0	

E. RESOURCES FOR WORK

The following question asks about the resources and supplies that you have for your work. For each, please tell me if you experience this **ALWAYS, OFTEN, SOMETIMES, RARELY, or NEVER.**

Only indicate one response for each declaration. If the declaration is not applicable (N/A), circle 0.

	**How often does the following affect your job performance in your community?**	**Always**	**Often**	**Some times**	**Rare ly**	**Neve r**	**N/ A**	
E1	Lack of medication for patients living with HIV	5	4	3	2	1	0	
E2a	Lack of prophylaxis for patients living with HIV	5	4	3	2	1	0	
E2b	Lack of other medicines	5	4	3	2	1	0	
E3	Lack of rapid test for HIV	5	4	3	2	1	0	
E4	Lack of PCR tests (for HIV)	5	4	3	2	1	0	
E5	Shortage of stationery – pen, notebook, forms	5	4	3	2	1	0	
E6	Shortage of other tools necessary for the job	5	4	3	2	1	0	
E7a	Lack of available transport during work	5	4	3	2	1	0	
E 8	Lack of lunch for health workers	5	4	3	2	1	0	
E 10	In general, how satisfied are you with the resources available at the health center you work in?	5	4	3	2	1	0	

F. SELF-EFFICACY AND FEEDBACK MECHANISMS

The following statements are intended to reflect the self-efficacy and ways that health workers receive feedback on their work. For each of the statements, please think about your work environment and HOW STRONGLY each of the statements captures your experiences. Please tell me if you **STRONGLY AGREE, AGREE, NEUTRAL, DISAGREE, OR STRONGLY DISAGREE.**

Only indicate one response for each declaration. If the declaration is not applicable (N/A), circle 0.

		Strongly Agree	Agree	Neutral	Disagree	Strongly Disagree	N/A	
F1	I feel confident that I am performing very well in my job.	5	4	3	2	1	0	
F2	I find it difficult to cope with the demands of the job.	5	4	3	2	1	0	
**I know I am performing my job well when…**								
F3	My supervisor tells me.	5	4	3	2	1	0	
F4	Change in supervisor's attitude/behavior.	5	4	3	2	1	0	
F5	Other *[type of health worker]* tell me	5	4	3	2	1	0	
F6	The community members tell me.	5	4	3	2	1	0	
F7	Change in the community beneficiaries knowledge, attitudes and behavior	5	4	3	2	1	0	
F8	The job becomes easier.	5	4	3	2	1	0	
F9	I just know it	5	4	3	2	1	0	
**I know I am not performing my job well when..**								
F10	My supervisor tells me.	5	4	3	2	1	0	
F11	Change in supervisor's attitude/behavior	5	4	3	2	1	0	
F12	Other *[type of health worker]* tell me.	5	4	3	2	1	0	
F13	The community members tell me	5	4	3	2	1	0	
F14	Change in the community beneficiaries knowledge, attitudes and behavior	5	4	3	2	1	0	
F15	The job becomes harder.	5	4	3	2	1	0	
F16	I just know it.	5	4	3	2	1	0	

G. Time Commitment: We want to understand how much time you spend on activities relating to your work.

N^O	QUESTIONS	
G1	How much time do you USUALLY spend doing administrative work **in a normal day**?	HOURS [___][___]<br>MINUTES [___][___]
G3a	How long do you USUALLY spend conducting **one** woman's **first** prenatal care consultation?	HOURS [___][___]<br>MINUTES [___][___]
G3b	How long do you USUALLY spend conducting **one** woman's **follow-up prenatal care consultation**?	HOURS [___][___]<br>MINUTES [___][___]
G3c	How long do you USUALLY spend conducting **follow-up prenatal care consultation of one woman living with HIV**?	HOURS [___][___]<br>MINUTES [___][___]
G3d	How long do you USUALLY spend attending to **one** woman giving birth?	HOURS [___][___]<br>MINUTES [___][___]
G3e	How long do you USUALLY spend for **one** child-at-risk consultation for an HIV-exposed child?	HOURS [___][___]<br>MINUTES [___][___]
G4a	How many patients do you see in **a normal day**?	[___][___]
G5	**In a month**, how many visits to the field do you make?	[___][___]
G6	In addition to birth consultations, administrative work and patient visits, how much time do you USUALLY spend doing other *[type of health worker]* related work in a month? (rephrased)	HOURS [___][___]<br>MINUTES [___][___]
In a normal week, how do these time commitments compare with the time you USUALLY spent on each of the following activities a year ago?		
G7a	First prenatal care visits	About the same .... 1<br>More time spent (now)............. 2<br>Less time spent (now) 3
G7b	Follow-up prenatal care visits	About the same .... 1<br>More time spent (now)............. 2<br>Less time spent (now) 3
G7c	Follow-up prenatal care visits with women living with HIV	About the same .... 1<br>More time spent (now)............. 2<br>Less time spent (now) 3
G7d	Attending a woman giving birth	About the same .... 1<br>More time spent (now)............. 2<br>Less time spent (now) 3
G7f	Child-at-risk consultation	About the same .... 1<br>More time spent (now)............. 2<br>Less time spent (now) 3
G8	Other health related activities besides administrative work, consultations, and attending births	About the same .... 1<br>More time spent (now)............. 2<br>Less time spent (now) 3
G9	Other personal activities	About the same .... 1<br>More time spent (now)............. 2<br>Less time spent (now) 3

I. Goal Setting: We would like to know your views on the goals that you have for Começando Saudável, how these goals were set, and how these goals influence your work.

	**ASKED AT ENDLINE ONLY**<br><br>For each of the statements, please think about your goals for Começando Saudável and HOW STRONGLY each of the statements captures your view. Please tell me if you strongly agree, agree, agree somewhat, disagree, or strongly disagree.<br><br>Again, please be reassured that all your responses are CONFIDENTIAL and no one from your work will have access this information.						
		Strongly Agree	Agree	Agree Somewhat	Disagree	Strongly Disagree	N/A
I1	Myself and my colleagues have specific, clear goals to accomplish for Começando Saudável.	5	4	3	2	1	0
I2	The goals for Começando Saudável are reasonably challenging (not too easy, not too hard).	5	4	3	2	1	0
I3	We have deadlines for accomplishing the Começando Saudável goals.	5	4	3	2	1	0
I5	I understand the reasons behind the Começando Saudável goals.						
I6	My supervisor is supportive and respectful in encouraging us to reach the goals.	5	4	3	2	1	0

I7	I participated in the setting of the Começando Saudável goals.	5	4	3	2	1	0
I8	My supervisor lets us participate in deciding how to reach these goals.	5	4	3	2	1	0
I9	We receive recognition when we attain the Começando Saudável goals.	5	4	3	2	1	0
I10	I get discouraged if we are not making progress toward these goals.	5	4	3	2	1	0
I11	Trying for the Começando Saudável goals makes my work more engaging than it would be without these goals.	5	4	3	2	1	0
I12	I feel proud when we get feedback indicating that we have reached the Começando Saudável goals.	5	4	3	2	1	0
I13	My colleagues and I encourage each other to reach the Começando Saudável goals.	5	4	3	2	1	0
I14	I sometimes compete with my colleagues to see who can do the best in working towards the Começando Saudável goals.	5	4	3	2	1	0
I15	I usually feel that we have a suitable action plan for reaching these goals.	5	4	3	2	1	0
I16	I feel that my colleagues and I are prepared for and capable of reaching the Começando Saudável goals.	5	4	3	2	1	0

I17	I feel that factors outside my control influence whether we achieve the Começando Saudável goals or not.	5	4	3	2	1	0
I18	Having the Começando Saudável goals help me to do my job well.	5	4	3	2	1	0
I19	There is pressure to achieve the Começando Saudável goals.	5	4	3	2	1	0
I20	I am concerned that pressure to achieve the Começando Saudável goals can lead to cheating and dishonesty among other colleagues.	5	4	3	2	1	0
I21	I keep the Começando Saudável goals in mind while I am working.	5	4	3	2	1	0

J. Incentives: We would like to know your views on the individual and group financial incentives you receive. ***FOR INTERVENTION HEALTH WORKERS ONLY, ASKED AT ENDLINE ONLY***

	For each of the statements, please think about your experience during Começando Saudável and indicate HOW STRONGLY each of the statements captures your view. Please tell me if you strongly agree, agree, agree somewhat, disagree, or strongly disagree.<br><br>Again, please be reassured that all your responses are CONFIDENTIAL and none of the supervisors will have access to your responses.						
N^{O}		Strongly Agree	Agree	Agree Somewhat	Disagree	Strongly Disagree	N/A
J1	Our (association, health center) can improve our delivery of services when we receive the group incentive.	5	4	3	2	1	0
J2	I felt discouraged if our (association or health center) does not receive the maximum incentive.	5	4	3	2	1	0
J3	The incentives help our (association or health center) work together to achieve the goal.	5	4	3	2	1	0
J4	I feel that that the amount of the incentives for our (association or health center) is sufficient.	5	4	3	2	1	0

J5	The group incentives make some colleagues lazy because they know others will do more of the work.	5	4	3	2	1	0
J6	Structuring the incentives to go to our (association or health center) is a good approach compared to just receiving personal incentives.	5	4	3	2	1	0
J7	I feel that the group incentives encourage us in the (association or health center) to support each other.	5	4	3	2	1	0
J8	The group incentives make everyone in the group work equally hard.	5	4	3	2	1	0
J9	I feel that the maximum amount of the individual incentive is sufficient.	5	4	3	2	1	0
J10	I can attend more patients and achieve my goals if I receive the individual incentive.	5	4	3	2	1	0
J11	I feel recognized when I receive an individual incentive.	5	4	3	2	1	0

Supplementary Material 2: SEMI-STRUCTURED EXIT INTERVIEW GUIDE

Time Started: ______ Time Completed:______

A1. Gender:_______

A2. Age:__________years

A3. Title of your position: ________________________________

A4. Length of time working in this position: ____________

A5a: Did you hold a related position before?

A5b: If so, what was it?

A5c. How long did you work in that other position? ____________

1. Please describe what you understand Começando Saudável, the PBF program, to be.
2. Please describe your role in Começando Saudável.

3.0 Think about the state of the health system (health center) at the time Começando Saudável was being developed and implemented (June-August 2013).

Please describe your experiences of the process of developing the PBF model.

3.1 Probes: What do you remember about deciding on the indicators, setting the goals for the indicators, group-level goals, designing quarterly evaluations, type of incentives, how funds would be dispersed).

3.2 What worked well in developing Começando Saudável?

3.3 What were some challenges that you experienced?

3.4 In other places, stakeholders have faced constraints when developing a program, such as feeling comfortable speaking their mind. Did you encounter any such problems?

3.5 What would you or your colleagues do differently when developing such a program in the future?

4.0 Do you think Começando Saudável was well understood by health workers?

4.1 Did the Começando Saudável affect the attitude of health workers? How?

5.0 How do you and your colleagues feel about the process of how the performance of you and your colleagues was evaluated(to see if goals were met)?

5.1 What was your opinion of the performance goals? Did you think the goals were reasonable? Do you wish they were somehow different? How?

5.2 How do you think having goals for the entire group (health center) worked?

Probes: Any positive effect? Which ones? Any negative effect? Which ones? Did it help workers collaborate towards the goal?

5.3 How do you feel the quarterly evaluation of performance went?

5.4 What would you recommend to improve the evaluation of achievement of the goals?

6.0 Were there any external factors that affected the performance of your (district, health center) for any of the quarterly evaluations?

6.1 What were they? Probes: Transition from Mais Vida to CCS as HIV prevention and treatment partner in the district. Major stock-outs of medicine. Turn-over of staff. Tensions among staff. When?

6.2 In light of these, do you have any recommendations for how Começando Saudável could/should have responded? How?

7.0 Please describe how you decided how funds would be used.

7.1 How do you feel that this process went? What would you recommend to improve this? How?

8.0 Please describe the process for receiving funds earned.

8.1 How do you feel that this process went?

8.2 What would you recommend to improve this?

8.3 We heard that there were challenges in disbursement of funds; that funds for the health center had to be transferred into the personal account of heatlh center workers. How do you feel about that? (Any concerns with transparency?)

9. Do you think that having these goals motivated your colleagues to do their job better? Worse? Do you think that receiving incentives motivated your colleagues to do their job better? Please explain.

9.1 Were the incentives appropriate to motivate health workers? Why or why not?

9.2 In your opinion, how did it work to have the payment of the individual incentives depend on attaining the group goals and receiving the group incentives?

10.0 Did Começando Saudável affect the overall delivery of maternal and child health and prevention of vertical transmission services in your district? How?

10.1 Do you think it helped increase community contacts of patients? How?

10.2 Do you think it affected retaining women and child in care at the health centers? How?

11.0 What relationship do activistas have with the district? Does it vary by health center? What makes it work well in health centres where activistas have strong linkages?

11.1 Activistas also are part of Começando Saudável, and they have goals to increase number of home visits with pregnant women who living with HIV and with children exposed to HIV. Have you noticed an change in number of partients comeing for PVT services due to activista involvement?

11.2 Have you noticed a change in collaboration among health centres and associations due to Comencando Saudavel?

12.0 How did the PBF affect your (district, health center)?

12.1 What were some positive effects? Probes: Facilitate the (district's, health center's) autonomy to prioritize funds? More resources? Changes in supervision? Collaboration among sectors?

12.2 What were some negative effects? Probes: In other PBF programs, people have reported prioritizing work to meet the PBF goals at expense of other work duties and falsifying reports. Have you or your colleagues heard of anything like this happening in your (district, health center)?

13. Começando Saudável is conducting our evaluation, and it looks like using performance-based financing did not have an effect on our indicators (number of PTV services delivered). Why do you think this happened, that the program was not successful?

14.0 How would you describe the relationship between the district and health centers and between Sede and the periphery? Any challenges?

14.1 Probe: During Começando Saudável, we observed challenges in communication and processes between district and health centers and between Sede and peripheral health centers in terms of ordering and distribution of supplies. Did you see anything like this?

14.2 How do you think this affected the PBF initiative?

14.3 Do you think other systemic issues need to be sorted out before trying a new PBF initiative? Like what?

15. What advice do you have for another district (health center) considering implementing a PBF?

For Comparison District only:

16. How do you feel about being the control district for *Começando Saudável*?

Thank-you for sharing your time and this helpful information.
